# Acute Obstructive Jaundice Secondary to Surgical Clip Migration Into the Common Bile Duct 12 Years Following Laparoscopic Liver Resection: A Case Report

**DOI:** 10.7759/cureus.89321

**Published:** 2025-08-04

**Authors:** Genki Watanabe, Sadatoshi Shimizu, Tomoaki Yamasaki, Akihiro Murata, Akishige Kanazawa

**Affiliations:** 1 Hepato-Biliary-Pancreatic Surgery, Osaka Metropolitan University, Osaka, JPN; 2 Hepato-Biliary-Pancreatic Surgery, Osaka City General Hospital, Osaka, JPN; 3 Gastroenterology, Osaka City General Hospital, Osaka, JPN; 4 Gastrointestinal and Pediatric Surgery, Tokyo Medical University, Tokyo, JPN

**Keywords:** acute cholangitis, complication, laparoscopic liver resection, obstructive jaundice, surgical clip migration

## Abstract

Surgical clip migration to the common bile duct is a rare late complication, typically originating from clips placed at the cystic duct and most commonly reported after laparoscopic cholecystectomy. We present an exceptionally rare case of obstructive jaundice caused by clip migration from the liver dissection plane, rather than from the cystic duct, occurring 12 years after laparoscopic liver resection (LLR) and cholecystectomy and associated with chronic biliary inflammation.

A 73-year-old man underwent LLR of segments 4a + 5 and cholecystectomy for hepatocellular carcinoma and was discharged on postoperative day 12 without any complications. Three months later, computed tomography revealed a fluid collection along the liver dissection plane and dilation of the bile duct of segment 3 of the liver (B3), both of which were followed up without intervention due to the absence of symptoms and significant abnormalities in laboratory data. Eight years later, he required percutaneous transhepatic biliary drainage (PTBD) from the B3 due to bile duct stenosis and recurrent cholangitis. He was followed up as an outpatient with regular PTBD tube exchanges. Twelve years after the LLR, he developed acute obstructive cholangitis caused by a high-density lesion in the distal bile duct. Endoscopic removal identified the lesion as a migrated surgical clip. Because the clips at the cystic duct stump remained in place, the migrated clip was identified as originating from the liver dissection plane. Since surgical clip migration to the common bile duct following cholecystectomy has been reported to result from inflammation around the cystic duct stump, the clip migration observed in this patient may also be associated with chronic inflammation along the liver dissection plane or the PTBD tract.

Chronic inflammation along the liver dissection plane may have led to the clip migration. Surgeons should be aware of the potential for clip migration from the liver dissection plane into the common bile duct as a rare long-term complication following LLR, especially in patients with chronic inflammation around the bile duct.

## Introduction

Clip migration is reported as a rare and long-term complication after open or laparoscopic cholecystectomy with clips placed around the stump of the cystic duct [1]. Various mechanisms of clip migration after cholecystectomy have been hypothesized, including inflammation around the cystic duct stump because of minor bile leakage caused by incomplete surgical clips and mechanical compression of adjacent surgical clips to the common bile duct [1,2]. Although these migrated clips were originally located near the stump of the cystic duct, no studies have reported clip migration into the bile duct originating from the liver dissection plane or percutaneous transhepatic biliary drainage (PTBD) tract.

Herein, we present a rare case of acute obstructive jaundice secondary to surgical clip migration from the liver dissection plane or PTBD tract into the common bile duct 12 years following laparoscopic liver resection (LLR) and cholecystectomy.

## Case presentation

A 73-year-old man with a history of hepatitis C virus infection underwent laparoscopic anatomical liver resection of segments 4a + 5 and cholecystectomy for hepatocellular carcinoma at our hospital. He was discharged from the hospital on postoperative day 12 without any complications. Three months later, a computed tomography (CT) scan revealed a fluid collection along the liver dissection plane and dilation of the bile duct of segment 3 of the liver (B3), both of which were followed up without intervention due to the absence of symptoms and significant abnormalities in laboratory data. Eight years after undergoing LLR, he developed bile duct stenosis of the B3 and recurrent cholangitis. Since endoscopic stent placement across the stricture was technically difficult, PTBD was performed, bridging the common bile duct from the B3. Using the PTBD catheter for internal drainage and regular exchanges, he received outpatient care after successful cholangitis management.

He was admitted to our hospital with high fever and fatigue at the age of 85. Laboratory study results revealed elevated inflammatory response and hepatobiliary enzyme levels (Table 1): white blood cell count of 12,740 cells/μL (normal range 3,580-8,150 cells/μL), hemoglobin of 12.5 g/dL (13.3-16.6 g/dL), platelets of 15.8×10^4^/μL (17.2-35.9×10^4^/μL), total bilirubin of 4.5 mg/dL (0.2-1.2 mg/dL), direct bilirubin of 2.0 mg/dL (0.0-0.4 mg/dL), aspartate aminotransferase of 201 IU/L (8-38 IU/L), alanine aminotransferase of 186 IU/L (4-44 IU/L), alkaline phosphatase of 235 IU/L (38-113 IU/L), γ-glutamyl transpeptidase of 419 IU/L (16-73 IU/L), lactate dehydrogenase of 296 IU/L (124-222 IU/L), creatinine of 1.37 mg/dL (0.60-1.10 mg/dL), C-reactive protein of 13.36 mg/dL (≤0.26 mg/dL), and prothrombin time of 96.2% (80%-127%). These data indicated acute obstructive cholangitis.

**Table 1:** Laboratory findings with normal reference ranges. WBC, white blood cell; Plt, platelets; T-Bil, total bilirubin; D-Bil, direct bilirubin; AST, aspartate aminotransferase; ALT, alanine aminotransferase; ALP, alkaline phosphatase; GGT, γ-glutamyl transferase; LDH, lactate dehydrogenase; Cr, creatinine; PT, prothrombin time; CRP, C-reactive protein

Laboratory test	Result	Normal range	Unit
WBC	12,740	3,580–8,150	cells/μL
Hemoglobin	12.5	13.3–16.6	g/dL
Plt	15.8	17.2–35.9	×10^4^/μL
T-Bil	4.5	0.2–1.2	mg/dL
D-Bil	2.0	0.0–0.4	mg/dL
AST	201	8–38	IU/L
ALT	186	4–44	IU/L
ALP	235	38–113	IU/L
GGT	419	16–73	IU/L
LDH	296	124–222	IU/L
Cr	1.37	0.60–1.10	mg/dL
PT	96.2	80–127	%
CRP	13.36	≤0.26	mg/dL

**Figure 1:**
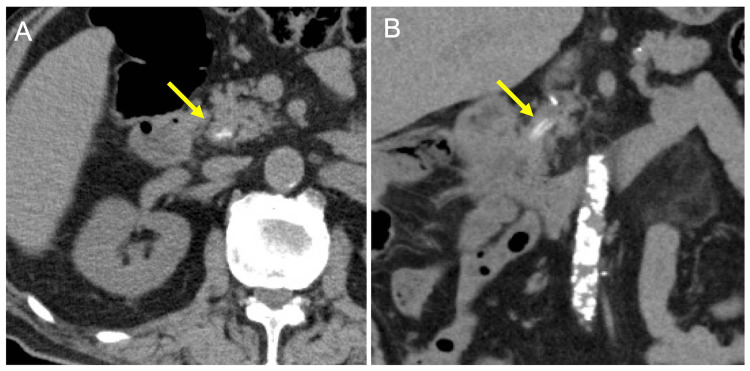
CT scan upon admission for obstructive jaundice. A CT scan at the level of the papilla of Vater reveals a high-density lesion in the distal bile duct (arrow) on the axial (A) and coronal (B) images. CT, computed tomography

**Figure 2:**
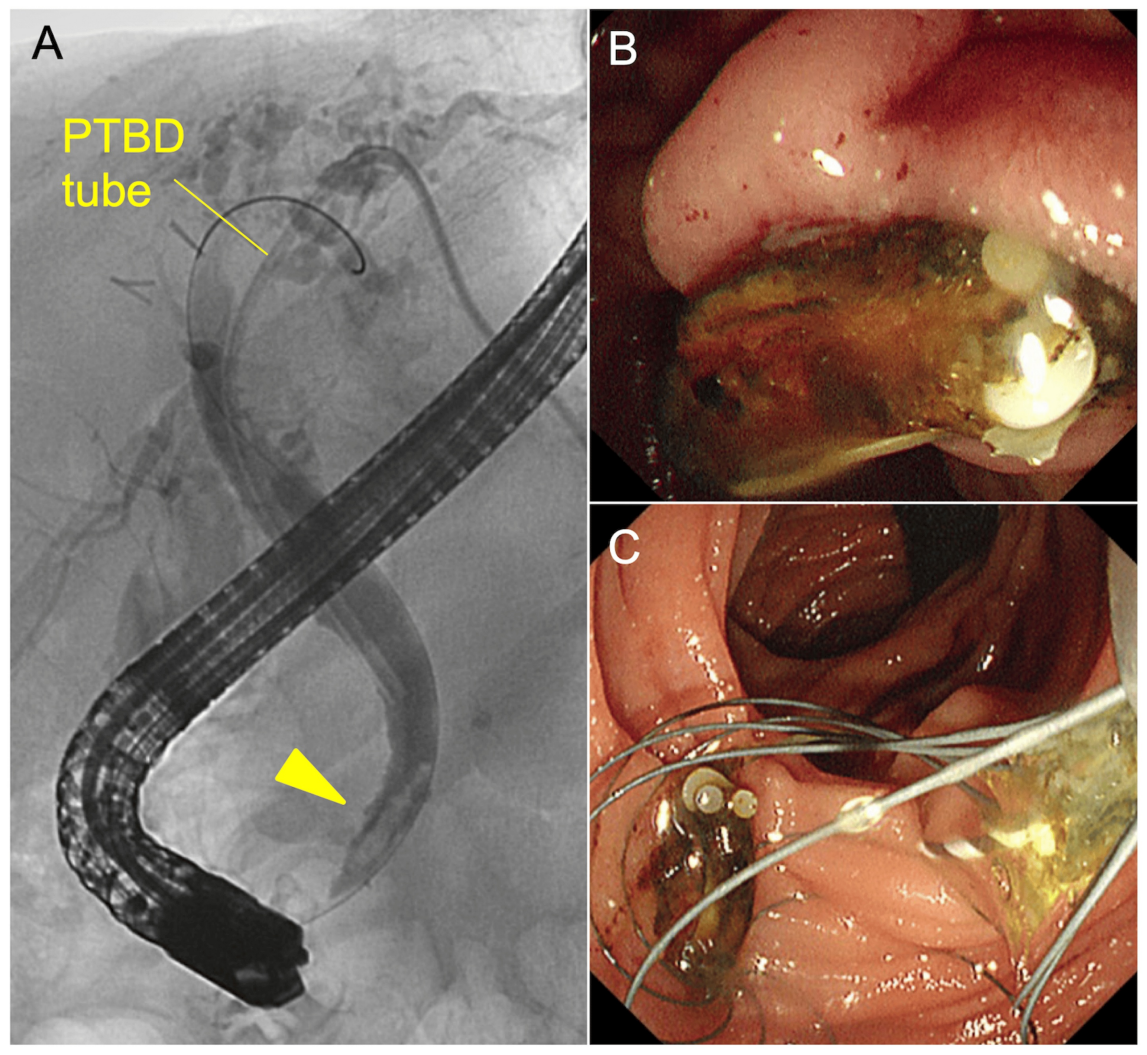
Endoscopic findings. A. Endoscopic retrograde cholangiopancreatography also detects a filling defect in the distal bile duct without radio-opacity (arrowhead). B, C. The obstructive lesion is removed and identified as a surgical clip (Hem-o-Lok, size ML). PTBD, percutaneous transhepatic biliary drainage

**Figure 3:**
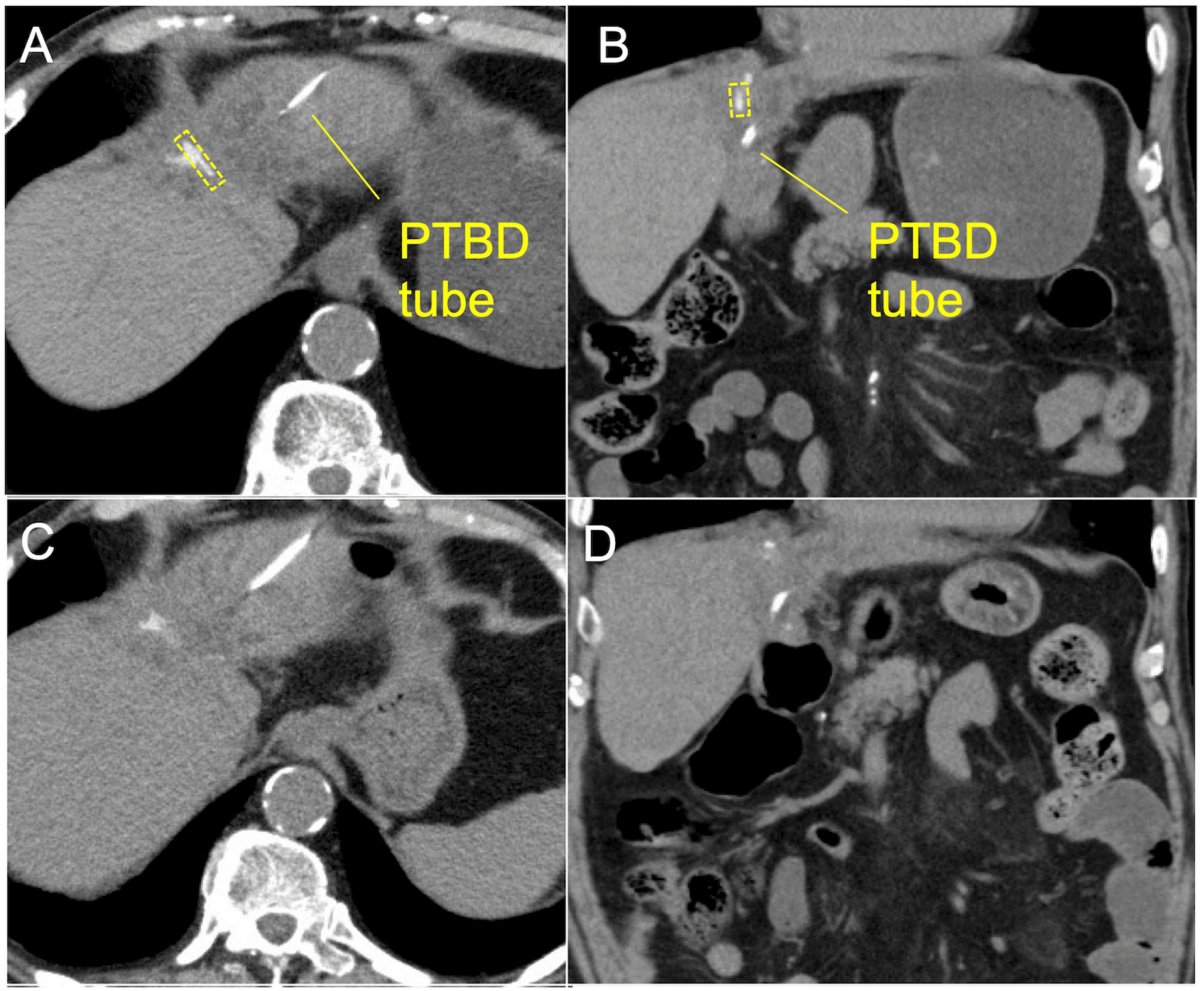
CT scans three months before and upon admission. A, B. A CT scan performed three months before admission at the level of the liver dissection plane shows a clip on the liver dissection plane (indicated by a dotted line). C, D. The clip is no longer visible at the same level on the CT scan obtained at admission. The left and right sides show the axial and coronal images, respectively. CT, computed tomography; PTBD, percutaneous transhepatic biliary drainage

**Figure 4:**
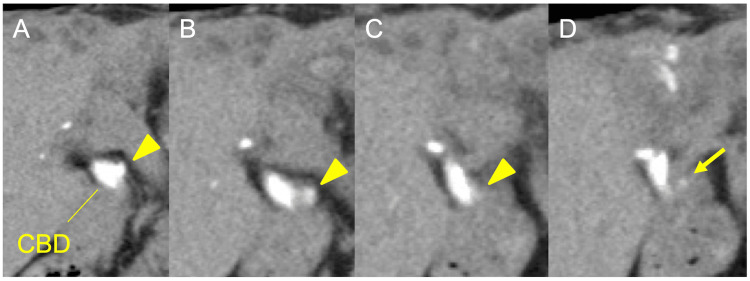
Surgical clip at the cystic duct stump. A-D. Based on the previous drip infusion cholangiography–computed tomography, the remnant cystic duct (arrowhead) is seen flowing into the common bile duct. A surgical clip (Hem-o-Lok, size M) marks the cystic duct stump (arrow). These coronal images are presented in consecutive slices. CBD, common bile duct

The CT scan revealed a high-density lesion in the distal bile duct (Figure 1) that had not been observed in the CT scan performed three months earlier. Endoscopic retrograde cholangiopancreatography also detected a filling defect in the distal bile duct without radio-opacity. The obstructive lesion was subsequently removed and identified as a surgical clip (Hem-o-Lok, size ML), not merely a common bile duct stone (Figure 2). Referring to the previous CT scan retrospectively, the CT scan upon admission revealed that a surgical clip around the liver dissection plane was missing (Figure 3). The clip at the cystic duct stump was located on the left side of the common bile duct (Figure 4). Furthermore, the size of the migrated clip was ML, which differed from the M-sized clip documented in the surgical record as having been used at the cystic duct stump during the LLR and cholecystectomy. These findings suggest that the clip may have migrated into the common bile duct from the liver dissection plane or PTBD tract rather than from the cystic duct stump. After managing the obstructive cholangitis, he was discharged on the fourth day following the removal of the migrated surgical clip.

## Discussion

Obstructive jaundice caused by clip migration is reported as a rare and long-term complication after cholecystectomy with clips placed around the stump of the cystic duct (being identified as the underlying cause) [1,3]. Our patient developed obstructive jaundice caused by surgical clip migration to the bile duct, which may have originated from the liver dissection plane or the PTBD tract. To the best of our knowledge, there have been no reported cases of clip migration from the liver dissection plane into the common bile duct. Surgeons should be aware that clip migration can occur after LLR in the presence of chronic inflammation around the bile duct, and it can lead to obstructive jaundice as a long-term complication.

Postcholecystectomy clip migration was first reported in 1979 following open cholecystectomy and in 1992 following laparoscopic cholecystectomy [4,5]. As the number of cholecystectomies has increased, reports of clip migration have also accumulated, with approximately 120 cases of clip migration into the bile duct reported between 1997 and 2021, regardless of the surgical approaches [1,3,6]. Among these patients, 70% underwent laparoscopic cholecystectomy [1,3,6]. According to these reviews, almost all the initial operation included cholecystectomy, and the clips at the stump of the cystic duct most frequently migrated into the bile duct. The median duration from surgery to presentation ranged from 24 to 49 months [1,3,6]. Clip migration more than 10 years after the initial operation has also been reported [3,7]. Endoscopic removal of the migrated clip was successful in 55-77% of cases [3]. Similarly, our case presented with acute obstructive cholangitis 12 years after the initial surgery and was successfully treated endoscopically.

Although the exact mechanism remains unclear, several hypotheses have been proposed regarding clip migration after cholecystectomy. These include inflammation around the cystic duct stump because of minor bile leakage caused by incomplete surgical clips and mechanical compression of adjacent surgical clips to the common bile duct [1,2,8]. Our patient did not develop any infectious complications during hospitalization, including bile leakage following LLR and cholecystectomy. However, mild inflammation at the liver resection plane such as bile leakage or biloma formation was suspected, as a CT scan performed three months postoperatively revealed fluid collection and intrahepatic bile duct dilatation. A PTBD tube was eventually inserted for recurrent cholangitis caused by bile duct stenosis, and the migrated clip was found near the PTBD tract. Therefore, chronic inflammation around the bile duct, combined with mechanical compression from the surgical clip that was presumably placed at the stump of the B4 and exerted pressure on the B3, may have contributed to its migration into the bile duct. Although inappropriate clipping during surgery may have played a role in the postoperative inflammation, this cannot be confirmed due to the lack of intraoperative video.

When cholecystectomy is performed simultaneously with LLR, the migrated clip is commonly reported to originate from the stump of the cystic duct [1,3,6]. Additionally, resection of liver segments 4a + 5 made it difficult to distinguish between the clip on the cystic duct stump and the clip on the liver resection plane because the cystic duct stump was close to the liver resection plane. However, in our patient, the origin of the migrated clip was different from the cystic duct. The surgical clip (Hem-o-Lok, size M) was located to the left of the common bile duct in the previous images, as confirmed by the operative record, whereas a surgical clip on the liver dissection plane was no longer visible compared to the CT scan performed three months earlier. These findings indicate that the surgical clip migrated into the bile duct might be located at the liver dissection plane or near the PTBD tract, not at the stump of the cystic duct.

To prevent surgical clips migration into the common bile duct, several measures have been proposed, including the use of absorbable clips or sutures instead of non-absorbable polymer Hem-o-Lok clips, and the retrieval of any incomplete clips [1,9-11]. The broad exposure of the hepatic hilum after LLR of segments 4a + 5 may facilitate the mechanical compression of the major bile duct by surgical clips. Therefore, the use of absorbable clips or stitches around the major bile ducts may be advisable during LLR, considering the potential for migration. With respect to suturing around the major bile duct, robot-assisted liver resection may offer greater advantages than LLR due to its ergonomic motion and enhanced joint articulation [12], which facilitate laparoscopic suturing during surgery.

## Conclusions

Surgeons should be aware of the potential for clip migration from the liver dissection plane into the common bile duct as a rare long-term complication following LLR, especially in patients with chronic periductal inflammation, such as that caused by bile leakage or biloma formation.
